# Phytochemicals Targeting Inflammatory Pathways in Alcohol-Induced Liver Disease: A Mechanistic Review

**DOI:** 10.3390/ph18050710

**Published:** 2025-05-11

**Authors:** Swati Tirunal Achary, Prerna Gupta, Apoorva Rajput, Wanphidabet Sohkhia, Srinivasa Reddy Bonam, Bidya Dhar Sahu

**Affiliations:** 1 Department of Pharmacology and Toxicology, National Institute of Pharmaceutical Education and Research (NIPER), Guwahati 781101, India; 2 Vaccine Immunology Laboratory, Department of Applied Biology, CSIR-Indian Institute of Chemical Technology, Hyderabad 500007, India; 3 Academy of Scientific and Innovative Research, Ghaziabad 201002, India

**Keywords:** alcohol, alcohol-induced liver diseases, immunological mechanisms, pro-inflammatory response, phytochemicals

## Abstract

Alcoholic beverages play a significant role in social engagement worldwide. Excessive alcohol causes a variety of health complications. Alcohol-induced liver disease (ALD) is responsible for the bulk of linked fatalities. The activation of immune mechanisms has a crucial role in developing ALD. No effective medication promotes liver function, shields the liver from harm, or aids in hepatic cell regeneration. Alcohol withdrawal is one of the most beneficial therapies for ALD patients, which improves the patient’s chances of survival. There is a crucial demand for safe and reasonably priced approaches to treating it. Exploring naturally derived phytochemicals has been a fascinating path, and it has drawn attention in recent years to modulators of inflammatory pathways for the prevention and management of ALD. In this review, we have discussed the roles of various immune mechanisms in ALD, highlighting the importance of intestinal barrier integrity and gut microbiota, as well as the roles of immune cells and hepatic inflammation, and other pathways, including cGAS-STING, NLRP3, MAPK, JAK-STAT, and NF-kB. Further, this review also outlines the possible role of phytochemicals in targeting these inflammatory pathways to safeguard the liver from alcohol-induced injury. We highlighted that targeting immunological mechanisms using phytochemicals or herbal medicine may find a place to counteract ALD. Preclinical in vitro and in vivo investigations have shown promising results; nonetheless, more extensive work is required to properly understand these compounds’ mechanisms of action. Clinical investigations are very crucial in transferring laboratory knowledge into effective patient therapy.

## 1. Introduction

Alcohol is a known and established liver toxin. The determination of safe limits for alcohol consumption remains a topic of ongoing debate [1]. As stated by the World Health Organization (WHO), having more than five standard drinks for males and four for females in 2 h can be known as binge drinking [2]. At the same time, the amount of alcohol consumed may not necessarily directly correlate with the severity of liver damage caused by alcohol [3]. Alcohol causes a wide range of pathology in the body, but the majority of associated mortality is caused by alcohol-induced liver disease (ALD). It is the most frequent cause of liver cirrhosis in Europe, America, Southeast Asia, and Central Asia [4]. According to WHO, hazardous alcohol use contributed to almost 30 lakh fatalities worldwide, which comprises 5.3% of all deaths. Globally, alcohol is the 7th most crucial risk factor for early mortality and disability [1]. Disability-adjusted life years (DALYs) are the number of years lost as a result of illness, disability, or premature death. It is the measure of the overall burden of the particular disease. India has the highest DALYs of alcohol-related liver cirrhosis, followed by the US, China, Nigeria, and Indonesia [5]. Men were observed to be more affected by fatalities due to alcohol compared to women [6]. Alcohol consumption can lead to a broad clinical spectrum of liver diseases, ranging from simple steatosis to cirrhosis and, ultimately, to fatal liver failure [7,8]. This process increases the risk of hepatocellular carcinoma [9].

The pathophysiology of ALD involves an intricate interplay between various mechanisms. The diverse clinical manifestations of ALD are furthermore complicated by genetic factors, dietary and metabolic factors, lifestyle, and immunological factors that make a person vulnerable to the course of the disease. The first condition that occurs due to excess alcohol use is alcoholic fatty liver (AFL). It is distinguished by the buildup of fats and triglycerides in more than 5% of hepatocytes. Some people will advance and develop alcoholic steatohepatitis, which is characterized by additional inflammation and hepatocyte damage [7]. The exposure to alcohol consumption determines the development of steatohepatitis, which can be reversed by abstinence [10]. Most people can develop liver steatosis after consumption of more than 60 g (76.04 mL) of alcohol each day for more than 2 weeks [11]. Some individuals may acquire alcoholic hepatitis and cirrhosis, the most severe form of ALD, if they continue to drink alcohol for a prolonged duration [10]. At the same time, the risk of cirrhosis rises even with lower alcohol intake levels. There is no clear-cut limit on alcohol intake deemed to be safe for the liver [11]. ALD is characterized immunologically by inflammation, featuring local and recruited inflammatory cells [9]. Inappropriate activation of resident immune cells, particularly Kupffer cells, is pivotal in the pathogenesis of ALD [12]. Alcohol-induced generation of free radicals and oxidative stress are also the main components influencing the progression of immunological consequences of ALD. However, new research has unequivocally demonstrated that the immunological response potentially plays a significant role in the onset of ALD, especially its inflammatory condition, alcoholic steatohepatitis [13].

Management of liver disease has become a daunting task. Alcohol abstinence must be the first step in treating ALD [14]. It is one of the most effective therapies for ALD patients. It can cause the reversal of steatohepatitis and increase the chances of survival of alcohol-induced cirrhotic patients [15]. It takes around 1.5 years of abstaining from alcohol to improve the overall survival of alcoholic cirrhosis patients [16]. There is no effective medication that promotes liver function and protects the liver from damage. The only medications available in the market are immunosuppressants and corticosteroids. These medications have numerous side effects. To name a few, pentoxifylline and prednisolone are recommended treatments for people with alcoholic hepatitis. Regretfully, many individuals do not react well to these medications [17]. Alcoholic steatohepatitis is a significant indicator of the progression of ALD. Corticosteroids have been the most researched and likely the most successful therapy option for steatohepatitis. However, the results of steroid therapies have been inconsistent [18]. Patients with severe alcoholic hepatitis who do not respond to corticosteroids can use pentoxifylline, a tumor necrosis factor (TNF)-α inhibitor [19], although there could be harmful side effects [20]. A liver transplant is the only option if the patient does not respond to the corticosteroid therapy [21]. In Western countries, ALD is the leading cause of liver transplantation, whereas hepatitis C virus (HCV) and non-alcoholic fatty liver disease (NAFLD) are the second and third leading reasons, respectively [22]. Similar patterns are also observed in Eastern countries. In India, ALD is one of the main signs of liver transplantation [23]. The current management of ALD with questionable safety and efficacy must be replaced with desirable candidates with minimal side effects. As a result, alternative, safe, and affordable treatment methods for ALD are desperately needed. Herbal medicine is one of the main sources of natural medicines, and it may be essential in developing hepatoprotective drugs [24]. Exploring natural products for ALD treatment that are inexpensive and have few adverse effects is a further field of research. Phytochemicals are abundant in bioactive constituents, which possess antioxidant, anti-inflammatory, and immunomodulatory properties [25]. Therefore, it is essential to learn more about the therapeutic role of these phytochemicals. Safer herbal remedies and having multiple targets have drawn greater interest in the past few years as an approach to prevent ALD.

**Figure 1:**
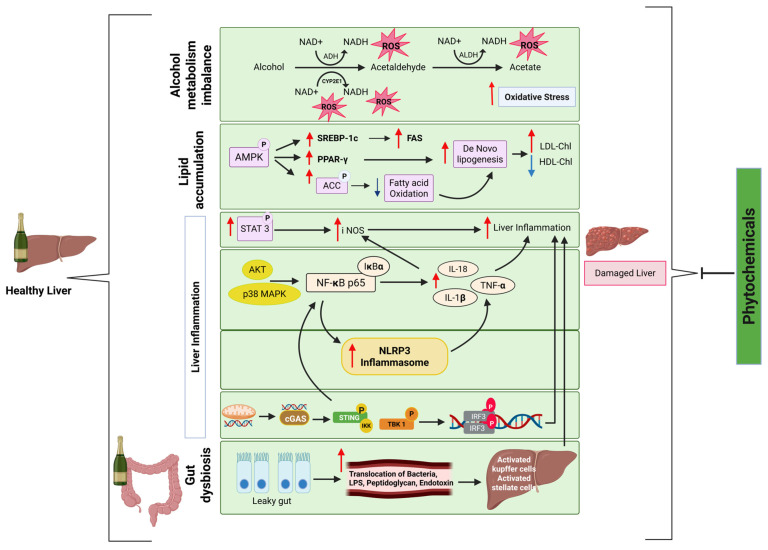
A diagrammatic overview of key pathways involved in ALD. This illustration outlines the principal signaling pathways activated during the progression of ALD. Chronic alcohol consumption disrupts the gut–liver axis, facilitates the translocation of endotoxins (e.g., LPS), and activates liver-resident immune cells, such as Kupffer cells, via TLR signaling. This activation subsequently triggers downstream signaling cascades, including the NF-κB, MAPK, and JAK-STAT pathways, leading to enhanced production of pro-inflammatory cytokines, increased oxidative stress, and sustained hepatic injury. Abbreviations: ADH, alcohol dehydrogenase; ALDH, aldehyde dehydrogenase; AMPK, AMP-activated protein kinase; SREBP-1c, sterol regulatory element-binding protein 1c; PPAR-γ, peroxisome proliferator-activated receptor gamma; FAS, fatty acid synthase; ACC, acetyl-CoA carboxylase; LDL-Chl, low density lipoprotein cholesterol; HDL-Chl, high density lipoprotein cholesterol. ↓: Downregulation; **↑**: Upregulation.

Despite several interesting existing reviews [26], this review is distinct in its specific focus on the immunological mechanisms driving ALD, and it systematically discusses how phytochemicals modulate key inflammatory pathways, such as Cyclic GMP-AMP Synthase–Stimulator of Interferon Genes (cGAS-STING), NOD-Like Receptor Family Pyrin Domain Containing 3 (NLRP3), Nuclear Factor kappa-light-chain-enhancer of activated B cells (NF-κB), Janus Kinase—Signal Transducer and Activator of Transcription (JAK-STAT), and Mitogen-Activated Protein Kinase (MAPK). It uniquely integrates the role of gut microbiota dysbiosis and innate immune responses in ALD progression, offering a mechanistic and translational perspective for future phytomedicine development. A diagrammatic overview of key pathways involved in ALD is depicted in Figure 1.

## 2. Inflammation-Associated Signaling Pathways in ALD and Its Modulation by Phytochemicals

### 2.1. Alteration of Intestinal Barrier Integrity and Gut Microbiota

**Figure 2:**
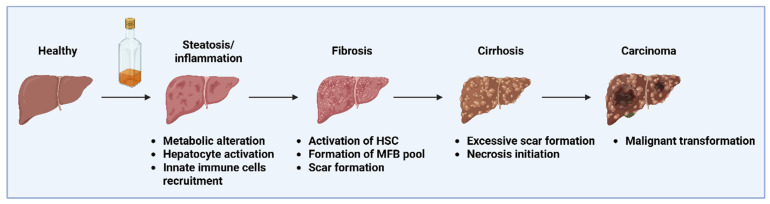
Stages of ALD. The illustration depicts the stage of the ALD from inflammation to hepatocellular carcinoma. Abbreviations: HSC, hepatic stellate cells; MFB, myofibroblasts.

The integrity of the intestinal barrier, control of gut homeostasis, and induction of the host immune response depend on the gut microbiota. ALD is influenced by impaired intestinal barrier integrity and the translocation of gut microbiota-derived products, which are transported from the gut to the liver through the gut–liver axis [27]. Chronic alcohol consumption alters the intestinal microbes’ makeup and encourages intestinal bacteria proliferation [28]. The gut–liver axis is one of the most probable initiators of inflammation in ALD. Long-term alcohol use damages the intestinal barrier, increasing intestinal permeability and stimulating the immune system ectopically. When gut homeostasis is maintained, several barriers shield the human body from microorganisms that invade it [29]. Both intestinal epithelial cells and the Paneth cells release antimicrobial proteins to prevent bacteria from migrating to the inner mucus layer, preserving the first physical barrier that separates the gut lumen from the host. Regenerating islet-derived 3 gamma (REG3G), a C-type lectin, can also be secreted by these cells to preserve the host and microbiota’s spatial segregation. Long-term alcohol consumption lowers intestinal REG3G expression, which is inversely correlated with the quality of bacteria associated with the mucosa in human patients [30]. Alcohol exposure lowers the amounts of tight junction proteins (such as occludin and ZO-1) and causes the death of epithelial cells at the tips of intestinal villi [31]. A crucial pathophysiological event underlying the change from alcoholic steatosis to alcoholic steatohepatitis is the translocation of bacteria or microbial products from the gastrointestinal mucosa to the liver [32]. Chronic alcohol consumption causes intestinal epithelium damage, increasing its permeability and providing a way for bacterial translocation from the intestine to the liver [33,34]. Various pathogens undergo liver infiltration, activating liver-resident monocytes to release pro-inflammatory cytokines [31]. Chronic alcohol consumption also causes an increase in the Gram-negative bacteria-led production of endotoxin that damages the integrity of the intestinal barrier. This process also increases the intestinal barrier permeability and reduces the bacteria responsible for the production of short-chain fatty acids [28]. Ethanol is metabolized in the liver with the help of different enzymes. Acetaldehyde is produced when alcohol dehydrogenase (ADH) oxidizes ethanol, and it is subsequently converted to acetic acid by aldehyde dehydrogenase (ALDH) [35,36]. Acetaldehyde, an ethanol metabolite, causes colonic epithelial injury and tight junction disruption [37]. Alcohol increases the expulsion of pro-inflammatory mediators like interleukin (IL)-1β and TNF-α in the small intestine and leads to intestinal inflammation that, in turn, causes intestinal permeability. This will increase the pathological bacteria translocation, increasing the plasma level of gut-derived microbes. Kupffer cells scavenge and phagocytose endotoxins to facilitate their elimination. However, when endotoxin accumulation exceeds the cells’ capacity, their phagocytic potential becomes overwhelmed, leading to endotoxin leakage into the bloodstream. Significantly higher levels of endotoxins are more prominent in ALD patients compared to normal individuals [38]. Lipopolysaccharide (LPS), peptidoglycan, etc., are the parts of gut microbes and will act as pathogen-associated molecular patterns (PAMPs) [39,40]. LPS attaches to its receptor, toll-like receptor (TLR)-4, in immune cells and other liver cells and starts an intracellular downstream signaling cascade. Further, free radicals induced via ethanol sensitize the hepatic stellate cells (HSC), and further endotoxin helps in their activation. HSC activation leads to extracellular matrix deposition, cytokine release, inflammation, etc., which worsen the alcoholic liver condition and progress toward chronic liver diseases [41,42] (Figure 2).

Several natural products show their protective effect against alcohol-induced gut microbiota dysbiosis and maintain the intestinal barrier integrity. These natural products include their bioactive compounds, extracts, or any other parts of the plant. Epigallocatechin-3-gallate (EGCG) is a phenolic compound. It inhibits gut leakiness and reduces endotoxemia caused by alcohol by blocking the activation of Kupffer cells. Endotoxins can trigger Kupffer cells to release a variety of inflammatory mediators, like TNF-α, through CD14/TLR4. EGCG suppresses the increase in both TNF-α and CD14 expressions in both serum and liver and alleviates ALD [43]. Lychee pulp, obtained from the plant *Litchi chinensis* Sonn., is rich in phenolic compounds. It attenuates the alcohol-induced liver injury via modulation of the activation of the endotoxin-TLR4-NFκB pathway. It increases the expression of mucus-protecting proteins and intestinal tight junction proteins and lowers the number of endotoxins in the blood [44]. Similarly, *Ginkgo biloba* and *Rosa roxburghii* of the family Rosaceae juice are rich in various bioactive compounds like quercetin, ginkgolide, rutin, and many more. They restore tight junctions, hence protecting against the intestinal barrier dysfunction that is caused by alcohol [45]. In another study, rice bran phenolic extract comprising derivatives of protocatechuic aldehyde, quercitrin, ethyl caffeate, and ethyl coumarate decreased pathogenic bacteria in the gut and protected the intestinal barrier, function, and permeability from alcohol. It alleviated the LPS and TLR4-mediated liver inflammation [46]. *Caulerpa lentillifera*, often called sea grapes, belongs to the green algae. It is an edible green seaweed with several nutritious and pharmacological benefits [46]. According to the studies, *Caulerpa lentillifera* treatment decreased alcohol-induced hepatic inflammation. *Caulerpa lentillifera*, when used as dietary supplementation in rats, reduced dysbiosis, and through the TLR4 pathway, it improved ethanol-induced liver damage, potentially slowing the course of ALD [47].

### 2.2. Hepatic Inflammation and the Role of Immune Cells in ALD

A multifactorial pathogenesis leads to the progression of ALD. Among these, the imbalanced immune-mediated functions cause chronic inflammation. Liver inflammation is mainly caused by gut-derived PAMPs, followed by the release of pro-inflammatory cytokines by the Kupffer cells and damage-associated molecular patterns (DAMPs) [7,48]. TLRs, a class of pattern recognition receptors (PRRs), can recognize both exogenous and endogenous PAMPs and DAMPs, which start an inflammatory cascade [49]. Chronic alcohol consumption causes gastrointestinal tract leaks and damages the intestinal barrier. As it becomes more permeable, the pathogens in the gut are more readily translocated into the circulation and lymphatic flow [50]. This causes the generation of microbial and toxic substances from the dying cells, called PAMPs/DAMPs, respectively. PAMPs enter the liver in this manner, where they stimulate the Kupffer cells, which, in turn, trigger the other immune cells that have infiltrated the liver [50]. It triggers the release of pro-inflammatory cytokines like TNF-α and IL-1β, further aggravating the ALD [51]. Cytokines, along with inflammatory mediators, contribute to the pathogenesis of the disease [52]. A wide range of cytokines that act in ALD are TNF-α, various ILs (IL-4, IL-6, IL-10, IL-1β), and interferon (IFN)-γ. In ALD, Kupffer cells are activated by ethanol-induced LPS, which produces inflammatory cytokines [53]. TNF-α is considered the major pro-inflammatory cytokine in the liver for alcohol-induced injuries [54,55]. In the liver, TNF-α is mostly generated by the activated Kupffer cells, and it also triggers the production of other cytokines. It has been shown that both the soluble and membrane-bound forms of TNF-α contribute to ALD [56]. It can bind to both receptors on the hepatocytes, TNFR-1 and TNFR-2. However, only TNFR-1 has a death domain capable of carrying out apoptosis directly. Contrarily, TNFR-2 lacks the death domain but is responsible for amplifying TNFR-1’s ability to induce both inflammation and cell apoptosis. TNFR-1 is crucial for hepatocyte proliferation because it activates the signal transducer and activator of transcription (STAT) 3 and NF-kB pathways [57]. Overall, ALD is characterized by increased production of pro-inflammatory cytokines [54,55]. Collectively, these cytokines attract inflammatory cells to the liver, kill hepatocytes, and start a healing reaction that involves scar tissue development and fibrosis [58] (Figure 2).

A typical healthy liver has a sizable population of localized immune cells that are distinct from the ones found in the peripheral circulation of the bloodstream. Kupffer cells, natural killer T (NKT) cells, certain antigen-presenting cells (APCs), and natural killer (NK) cells are among them. These specific immune cells are crucial for liver immune-mediated homeostasis. NK cells and NKT cells, for example, engage in the inhibition of liver metastases. Ethanol-fed mice exhibited a marked decrease in hepatic NK cell function [59]. NKT cells, which releases IL-10 upon activation, inhibit the protective action of NK cells in cases of ALD, such as steatohepatitis [60]. Both NK and NKT cells are innate immunity cells. Furthermore, ethanol-induced inhibition of precursor B-lymphocyte differentiation results in a decrease in the B-lymphocytes, which affects the humoral defense system in ALD patients [61]. In ALD, TLRs are abundantly expressed. Studies reported that an increase in the expression of TLR2 and a decrease in TLR3 expression activate STAT3 and lead to the generation of IL-10, which further promotes macrophage differentiation and, hence, mitigates ALD [62]. Macrophages play a crucial role in innate immunity. Its activation is crucial for immunological defense, responses to inflammatory agents, tissue repair, and homeostasis. Hepatic macrophages comprise both resident macrophages, known as Kupffer cells, and infiltrating macrophages. Collectively, they represent approximately 90% of the total macrophage population in the human body [63]. Kupffer cells are the first to receive signals and respond to invading hepatotoxic substances like alcohol by differentiating into various phenotypes to release anti-inflammatory factors. Simultaneously, they recruit many other macrophages, including Kupffer cells and circulating monocytes, which have similar functions and plasticity to that of Kupffer cells, into the liver [64]. Consequently, in a study, it is documented that gadolinium chloride (GdCl3) treatment leads to the inactivation of Kupffer cells and reduces damage in alcohol-induced liver disease, thus proving its role [41]. Macrophage polarization is the differentiation of macrophages into various phenotypes suitable for the particular microenvironment and condition when they are stimulated by particular stimuli, inflammatory agents, cytokines, or a pathogen [65]. According to Voican et al. [66], an increase in M2 polarization of macrophages and a decrease in infiltration of macrophages in adipose tissue were observed in alcohol withdrawal cases of ALD patients. In the progression of ALD, M2 macrophages appear to aid in the improvement of ALD by promoting hepatocyte senescence via IL-6 and preventing ALD [67]. Consuming alcohol also encourages neutrophils to infiltrate the liver, which accentuates the inflammatory process, encourages hepatic cell damage, and could be the cause of alcoholic hepatitis (Figure 2) [68].

As mentioned earlier, ALD is associated with activation of several inflammatory signaling pathways. Various innate immune cells facilitate the pro-inflammatory environment, including neutrophils, monocytes, macrophages, and others [69]. Neutrophil infiltration has become the hallmark of ALD and plays a crucial role in ALD progression, despite its protective role [70]. In ALD, multiple CXC chemokines mediate the migration of innate immune cells, such as interleukin (IL)-8 (CXCL8), CXCL6, CXCL1, CXCL10, and CXCL5. Hepatocytes and Kupffer cells secrete the said chemokines [71]. Neutrophils are the first innate immune cells to migrate to the environment. A recent study demonstrated that IL-8^+^ neutrophils are specifically enriched in the livers of patients with ALD, but not in their systemic circulation [72,73]. IL-8 is a key chemokine responsible for the recruitment and activation of neutrophils, and it is significantly elevated in individuals with ALD and correlates with disease severity [73]. Although rodents do not possess a direct homologue of IL-8, they produce cytokine-induced neutrophil chemoattractant-1 (CINC-1), an IL-8 analogue, in response to ethanol exposure [74]. Neutrophils interact with platelets via P-selectin–PSGL-1 binding, forming platelet–neutrophil aggregates that promote neutrophil activation and the release of neutrophil extracellular traps (NETs), which exacerbate liver inflammation and tissue damage [75]. In addition to neutrophils, other innate immune cells are recruited into the site, including monocytes, macrophages, NK cells, and others, followed by the recruitment of adaptive immune cells [71], which cumulatively contribute to the development of ALD. ALD encompasses a wide range of liver conditions, starting from simple alcoholic fatty liver (steatosis) and progressing to more severe forms such as steatohepatitis, fibrosis or cirrhosis, and hepatocellular carcinoma. These stages are typically identified based on liver histology in affected individuals [76]. However, the pathological features often overlap, rather than representing clearly separate disease stages [76].

In ALD, alcohol consumption increases the movement of endotoxins, such as LPS, from the gut to the portal circulation and activates the Kupffer cells via binding to TLR4. Another aspect of TNF-α activation is that it increases the metabolism in the hepatocytes, which results in reactive oxygen species (ROS) production. The LPS/TLR4 signaling and the ROS generation further activate the NF-кB signaling axis to exacerbate the tissue injury in the liver and hepatocyte apoptosis/necrosis. NF-kB activity regulates JNK activation. TNF-α consistently causes JNK activation when NF-kB is not activated. This prolonged JNK activation by TNF-α leads to cell death [77]. PAMPs act on the TLR4 and activate the NF-kB, releasing CC-chemokine ligand-2 (CCL2) and IL-8, which, in turn, trigger neutrophil and macrophage infiltration of the liver [50]. In contrast, DAMPs, including uric acid, ATP, adenosine, and DNA, are released during sterile inflammation and are responsible for cell death and loss of cell integrity. PAMPs, DAMPs, and IL-1β can act on the TLR4 receptor, activating NF-kB further and NLRP3 inflammasome signaling axis to precipitate inflammation. In addition, alcohol-induced liver injury also activates HSC, leading to cell proliferation that promotes the transforming growth factor-β (TGF-β) secretion. This can aggravate collagen synthesis and deposition of extracellular matrix components, leading to fibrogenesis. Furthermore, IL-1β activates the HSC through matrix metallopeptidase 9 (MMP9), intensifying liver fibrosis [78]. Studies have shown that sea buckthorn fermentation liquid showed a protective effect against alcoholic fatty liver disease. It downregulates the NF-kB and MAPK pathway by suppressing the TGF-beta activated kinase 1 (TAK1) activation. It also reduces the level of TNF-α and inhibits hepatocyte apoptosis [79]. A combination of taurine, EGCG, and genistein has also been studied for the intervention in liver fibrosis caused by alcohol via modulating inflammatory cytokines. Treatment with the combination compounds has restricted the production and secretion of inflammatory cytokines like IL-6 and TNF-α. Taurine promotes HSCs apoptosis via lowering *bcl2* mRNA expressions and suppressing TGF-β1 and Smad pathways. EGCG can inhibit collagen formation due to its potent antioxidant properties [80]. Whereas genistein can control the growth of liver sinusoidal endothelial cells by acting as a tyrosine kinase inhibitor. The combination therapy also promotes HSCs deactivation by significantly lowering the elevated levels of TGF-β1 and Smad3 [81]. Ginsenoside Rb1 (Grb1), a triterpene saponin (a glycoside), is isolated from *Panax quinquefolium* L. A study reported that Grb1 reduces the synthesis of pro-inflammatory cytokines, such as IL-1β and TNF-α, by inhibiting NF-kB expression. It considerably alleviates liver steatosis by lowering triglyceride levels and hepatic lipid accumulation. It prevents hepatic neutrophil infiltration, which is increased due to chronic alcohol consumption, thereby limiting further damage due to protease release and oxidative stress. Inhibition of neutrophil infiltration and decreased expression of the potent pro-inflammatory markers make it a strong candidate to stop alcohol-induced liver damage [82]. Myricetin, a polyhydroxyflavonol compound, is found in large quantities in fruits and vegetables. Myricetin may be an effective phytochemical, promising to be a potential candidate in alleviating ethanol-induced liver injury by reducing oxidative stress and mitigating inflammation. Reports suggest myricetin curbs the expression of inflammatory mediators (NF-κB and TNF-α, IL-6, IL-1β), and also restores the antioxidants, thereby protecting the liver from ethanol-induced damage [83]. In addition to the above, recent years of evidence have also documented that several other inflammatory signaling mechanisms contribute to ALD progression. In these inflammatory pathways, various phytochemicals have also been identified that alter the pathway and prevent the progression of the disease [84].

### 2.3. cGAS-STING Signaling in ALD

**Figure 3:**
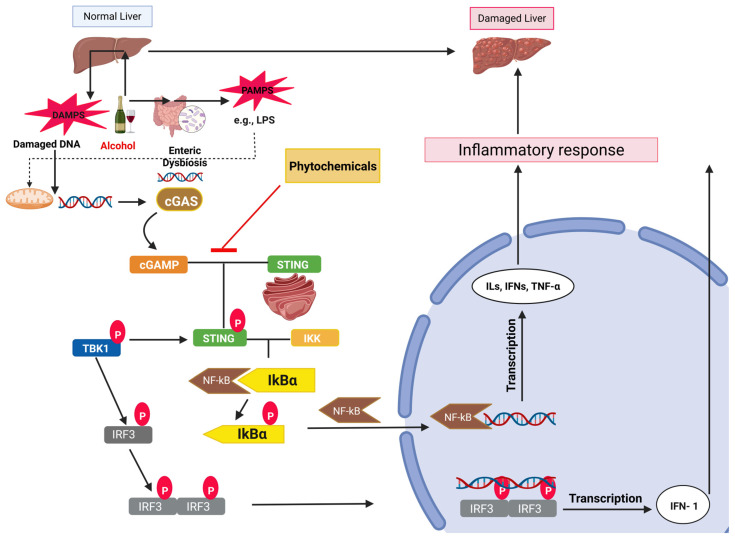
A diagrammatic representation of the cGAS-STING signaling axis involved in ALD. Alcohol and its metabolic byproducts promote the release of DAMPs, such as mitochondrial DNA, and PAMPs, such as LPS (known inducer of mitochondrial DNA release). These molecules are recognized by cyclic GMP-AMP synthase (cGAS) in the cytosol. Upon activation, cGAS stimulates the adaptor protein STING, which subsequently activates TBK1 and IRF3. This signaling cascade culminates in the induction of type I IFNs and pro-inflammatory cytokines, both of which contribute to liver inflammation and injury. Notably, certain phytochemicals have been shown to inhibit this pathway, thereby attenuating inflammation and hepatic damage.

cGAS is recognized as a direct sensor of cytoplasmic dsDNA and PRRs. The binding of c-GAS to dsDNA activates the cGAS-STING signaling, leading to the expression of type I IFNs and different inflammatory cytokines associated with innate immune responses [85]. This pathway is involved in the development of multiple liver diseases [86]. This innate immune response results in the activation of interferon regulatory factor 3 (IRF3), which increases the severity of ALD [87]. Alcohol consumption increases the liberation of cytoplasmic mitochondrial DNA (mtDNA), which initiates the cGAS-IRF3 signaling. Alcohol-induced activation of this signaling axis causes liver inflammation and injury in the hepatocytes and in the parenchymal cells residing with the hepatocytes by a gap junction intercellular communication pathway [87]. Also, the alcohol-induced hepatocytes undergo apoptosis after the activation of IRF3. Alcohol-induced cell damage produces DAMPs like damaged mtDNA and releases PAMPs like LPS, which then interact with the PRRs. cGAS has three dsDNA-binding sites in its structure and can detect canonical B-form DNA without any sequence specificity [88]. When cGAS molecules recognize dsDNA, they cross-link with one another to form dimers or multimers, causing the activation of cGAS [89,90]. Using ATP and GTP as substrates, cGAS catalyzes the cyclization of linear 2′-5′-linked dinucleotides and then the 3′-5′-phosphodiester linkage [91]. STING, an adaptor that resides in the endoplasmic reticulum (ER), changes its conformation upon binding to 2′, 3′-cGAMP and produces oligomers of STING [92,93,94]. Further, through the ER-Golgi intermediate compartment, the STING oligomer is transported to the Golgi [95,96]. In the signaling domain, TANK-binding kinase 1 (TBK1) is trans phosphorylated as a result of STING oligomerization upon ligand interaction [97]. In this process, phosphorylated TBK1 further phosphorylates IRF3 and STING. IRF3 dimerizes and moves into the nucleus, where it starts the IFN-1 transcription process [98,99]. Furthermore, STING attracts IкB kinase (IKK), which phosphorylates IкBα and causes NF-кB to migrate to the nucleus, where it transcribes a variety of cytokines, including ILs, IFNs, and TNF-α, initiating the inflammatory response and tissue damage in the liver [100,101]. The alcohol-induced cGAS-STING axis is shown in Figure 3. Considering the above facts, targeting the cGAS-STING pathway is a potential goal to alleviate ALD. However, the therapeutic benefits of phytochemicals or herbal products have not yet been explored against ALD by targeting this pathway. In this regard, further research is warranted. A flavonoid compound, Oroxylin A (OA), is one of the active ingredients of *Scutellaria baicalensis*. Its therapeutic properties have been explored in liver fibrosis. Oroxylin A inhibits the cGAS-STING pathway and induces the ferritinophagy of HSC. It inhibits HSC activation by inducing its senescence through ferritinophagy, which is a type of selective autophagy process [102].

### 2.4. NLRP3 Inflammasome Signaling Axis in ALD

Chronic alcohol consumption and its metabolic conversion lead to gut barrier disruption, resulting in the leakage of PAMPs, such as lipopolysaccharides (LPS), along with cellular contents, including adenosine triphosphate (ATP), high mobility group box 1 (HMGB1), and uric acid [36,103]. Also, the regular intake of alcohol activates the enzyme cytochrome P450 2E1 (CYP2E1) in the hepatocytes, which results in the massive production of ROS and causes stress in the endoplasmic reticulum and activates inflammatory responses via various pathways [36,104,105]. This further causes the generation of DAMPs. Now, these DAMPs, PAMPs, and leaky gut microbiomes further modulate the intracellular transduction axis and activate the inflammasome/NLRP3 signaling via PRRs in hepatocytes or Kupffer cells. This NLRP3 inflammasome sensor comprises the NLRP3, apoptosis-associated speck-like protein (ASC), and pro-caspase 1 [106]. The activation of NLRP3 inflammasome and caspase 1 leads to the cleavage of pro-inflammatory cytokines, i.e., pro-IL-18 and pro-IL-1β, into their active form, i.e., IL-18 and IL-1β, causing the inflammatory response in the liver [107]. Along with DAMPs and PAMPs, the ROS that are generated during alcohol metabolism also play a significant part in activating the NLRP3 inflammasome signaling. The imbalance between ROS production and their detoxification via antioxidant defense mechanisms, including superoxide dismutase (SOD), reduced glutathione (GSH), catalase, and many more, instigating oxidative stress in the liver [108]. The rise in oxidative stress in the liver further damages the mitochondrial DNA and produces more ROS. Mitochondrial ROS play a part in secondary stimulus and activate pro-caspase 1 into caspase 1 and also produce pro-inflammatory cytokines, including IL-18 and IL-1β. This activated caspase 1 again with the help of NLRP3 and ASC oligomerizes to form NLRP3 inflammasome, triggering further tissue damage in the hepatic tissues [109,110]. This NLRP3 inflammasome not only activates pro-IL-18 and pro-IL-1β but also cleaves gasdermin D (GSDMD), a protein with a significant role in the innate immune defense system against various PAMPs and DAMPs. The cleavage of GSDMD generates N-terminal fragments, which oligomerize within the plasma membrane to form pores secreting the activated IL-18 and IL-1β [111,112,113]. The pore formation in the membrane alters the integrity of the plasma membrane and causes a lytic form of cell death, i.e., pyroptosis [113,114]. There is some documented evidence to justify that the NLRP3 inflammasome is a viable target for ALD, and phytochemicals/herbal products can mitigate it. LanGui tea is a traditional Chinese medicine; its formulation comprises different herbs, which are *Gynostemma pentaphyllum*, *Cinnamomum cassia*, and *Ampelopsis grossedentata*. *G. pentaphyllum* has demonstrated potential in reducing inflammation, fatty liver, and liver steatosis [115]. Additionally, cinnamon helps lower hepatic steatosis and improve hyperlipidemia. These exhibit various protective properties against oxidative stress, inflammation, liver damage, and many other conditions. In ALD, it inhibits the NLRP3 signaling and reduces the generation of IL-1β [116]. Quercetin is a flavonoid of polyphenols. It has antioxidant properties and shows beneficial effects on alcohol-induced acute liver injury. It inhibits the ROS/NF-кB/NLRP3 axis by inducing IL-10 and heme oxygenase (HO)-1 [117]. Cannabidiol is extracted from marijuana plants without its psychoactive activity. Its effect has been studied in the ethanol plus high-fat diet model, in which it has been concluded that the compound inhibits the recruitment of macrophages and also impedes the NLRP3-pyroptosis pathway [118]. Dihydroquercetin is also known as taxifolin, which is a dihydroflavone. It is most abundantly found in onions, milk thistle, and other fruits. Taxifolin inhibits P2X7R signaling and IL-1β secretion by inactivating the NLRP3 inflammasome pathway. It also decreases caspase-1 activity [119]. Hence, exploration of NLRP3 inflammasome may be a viable target for the alleviation of ALD. Daucosterol, a phytosterol glycoside isolated from *Sanchezia spesiosa*, has been studied for ALD for its potential health benefits [120]. It also exhibits hepatoprotective activity in the carbon tetrachloride (CCl4)-intoxicated rat liver slices [121]. By modulating the p38 MAPK/NF-κB/NLRP3 inflammasome axis, daucosterol reversed the ethanol-induced oxidative damage, reduced lipid buildup, and mitigated hepatic inflammation. It reduces the alcohol-induced overexpression of the lipid synthesis genes fatty acid synthase (*FASN*) and sterol regulatory element-binding protein 1c (*SREBP1C*). It also reversed the alcohol-induced upregulation of collagen (COL)1A1, COL3A1, and α-smooth muscle actin (SMA). Moreover, it attenuated the alcohol-induced oxidative damage by restoring the hepatic antioxidants [122]. Ginsenoside Rk2 is one of the ginsenosides, a dehydro-protopanaxadiol saponin that has a strong anti-inflammatory profile and inhibits NLRP3 inflammasome activation [123]. It indicated a substantial decrease in the levels of triglycerides, aspartate aminotransferase (AST), and alanine aminotransferase (ALT) in the serum and showed hepatoprotective activity. Rk2 mitigates hepatic oxidative stress by promoting the nuclear factor erythroid 2–related factor 2 (Nrf2)/heme oxygenase-1 (HO-1) pathway. It also exerts anti-inflammatory action by blocking the NF-kB/NLRP3 inflammasome signaling pathway [124]. Scutellarin, an active glycosylated oxy-flavonoid component, is isolated from *Erigeron breviscapus* [125]. The defensive action of scutellarin toward ALD was reported. Alcohol-induced mRNA levels of pro-inflammatory mediators pertinent to the inflammatory response, such as IL-6, IL-1β, TNF-α, and iNOS, were substantially decreased by scutellarin action. In addition, scutellarin significantly reduces the degradation of IkBα and the increase of p-NF-κB (p65) and impedes the NLRP3, caspase-1, and ASC protein expressions in the liver [126].

### 2.5. NF-кB Signaling Axis in ALD

**Figure 4:**
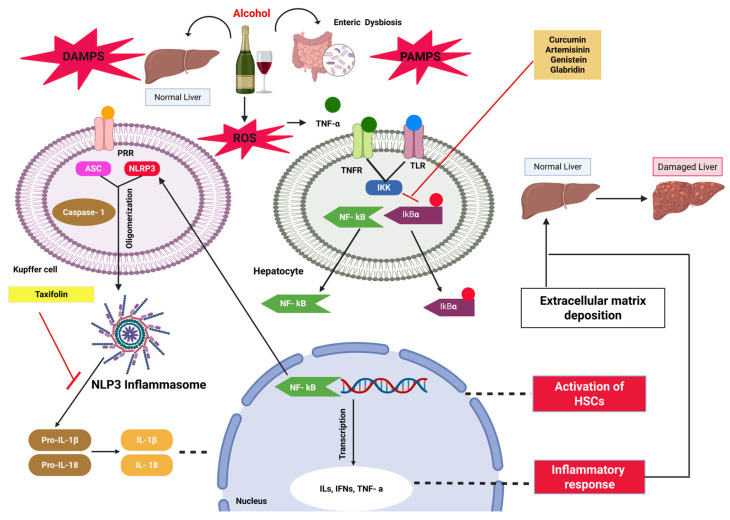
The diagrammatic representation of the NF-κB and NLRP3 inflammasome signaling pathway in ALD. Alcohol exposure increases intestinal permeability, facilitating the translocation of DAMPs and PAMPs into the liver, where they activate pattern recognition receptors, such as TLR4. This activation initiates downstream signaling cascades, including NF-κB activation and NLRP3 inflammasome assembly. Consequently, pro-inflammatory cytokines, such as IL-1β and TNF-α, are secreted, contributing to hepatic injury. Certain phytochemicals, including curcumin, artemisinin, and glabridin, have demonstrated the ability to modulate these pathways by inhibiting NF-κB translocation or NLRP3 inflammasome activation, thereby holding promise as potential therapeutic agents for ALD.

There are multiple inflammatory pathways associated with liver injury during ALD. One of the inflammatory pathways that trigger the immune cells of the liver is NF-kB signaling. The NF-kB protein family structurally contains five conservative members: NF-kB1 (p50), NF-kB2 (p52), RelA (p65), RelB, and c-Rel [127]. These proteins reside in the cytosol, and after the activation via various stimuli, they will form homo- or heterodimers and translocate in the nucleus and then bind with the respective DNA targets. In the cytosol, they are inactivated via molecular inhibitors of the NF-kB (IkB) family and activated by an inhibitor of kappa B kinase (IKK) [128,129]. Liver innate immunity plays a key role in the initial line of immunological defense against pathogens or endogenous danger signals. This immune system is governed by PRRs expressed on macrophages, epithelial cells, and others [129,130]. One of the receptors of PRRs is TLRs. After cellular damage due to ROS generation via ethanol-metabolized byproducts, other PAMPs/DAMPs contribute to activating the PRRs. The damaged cells also secrete inflammatory cytokines like TNF-α. This cytokine and the binding of LPS to the TLRs activate the NF-kB signaling cascade and the IKK kinase complex. This IKK phosphorylates IkB, which is then ubiquitinated and degraded via the proteasome pathway [131,132]. This process releases and activates NF-кB, which then moves to the nucleus, where it binds to DNA at a specific sequence, which further leads to the expression of different inflammatory factors. This NF-kB activation further propels the NLRP3 inflammasome, which then participates in inflammation and liver damage [133,134]. Many phytochemicals have shown hepatoprotective activity during ALD by modulating the NF-kB axis. Europinidin, an o-methylated derivative of delphinidin, is obtained from the plant *Plumbago Europaea* from the family Plumbaginaceae. It has been shown that the flavonoid europinidin improves liver health in rats. In this study, the intervention of europinidin restores the hepatic antioxidants and decreases lipid peroxidation in the liver tissue. Further studies also identified that the alcohol-induced pro-inflammatory cytokines (TNF-α, ILs, IFN-γ, and TGF-β) were substantially reduced in the europinidin treatment group and also interfered with the NF-kB signaling axis [135]. Turmeric contains curcumin, a naturally occurring polyphenol with strong anti-inflammatory and antioxidant properties. Studies reported that curcumin protects against liver damage due to ethanol consumption. The findings further suggest that curcumin regulates the ethanol-metabolizing enzyme CYP2E1 and also modulates the IkBα-NF-kB signaling axis to decrease inflammation in the liver [136]. Similarly, artemisinin has shown a cytoprotective effect in ALD. Artemisinin is obtained from *Artemisia annua* L. It is a sesquiterpene lactone endoperoxide, which suppresses nitric oxide (NO) synthase production and reduces the role of the transcription factor NF-kB. It also inhibits its activation and reduces the expression of inflammatory cytokines [137]. In another study, grape leaf extract, which is abundant in phenolic compounds and isolated from leaves of *Vitis vinifera* L., has alleviated alcohol-induced liver injury in rats. This extract improved the antioxidant defense system in the liver. The antioxidant and potential protective effect of this extract is probably mediated by the phenolic constituents found in it, such as derivatives of apigenin, epicatechin, quercetin, caffeic acid, and rosmarinic acid. Also, it inhibited the production of the pro-inflammatory cytokine (TNF-α) and expression of the NF-kB (p65) subunit in the liver [138]. Glabridin is an isoflavone derived from the roots of *Glycyrrhiza glabra* L. It improves liver injury by modulating the NF-кB pathway, reducing its nuclear translocation, and decreasing inflammatory cytokines [139]. Genistein is isolated from *Hydrocotyle sibthorpioides* Lam., a folk medicine in China. It has been studied for its antioxidant effect. It protects against alcohol-induced liver damage by reducing the DNA binding activity of NF-kB and its downregulation and the inhibition of inflammatory cytokines release [140]. Apigenin (4′, 5, 7-trihydroxyflavone) is a flavonoid compound found in fruits and a variety of medicinal plants. It exhibits hepatoprotective activity. It acts as a peroxisome proliferator-activated receptor alpha (PPARα) receptor agonist, and it also increases PPARα expression. The increased expression of PPARα downregulates the NF-kB signaling, hence reducing the inflammation of hepatic cells [141]. In *Carthamus tinctorius* L., hydroxysafflor yellow A (HSYA) is a primary chemical component, and its structure constitutes a solitary chalcone glycoside component. It has a strong antioxidant and anti-inflammatory profile [142,143]. Studies suggested that HSYA contributed significantly to the regulation of STAT3/NF-kB and phosphoinositide 3-kinase (PI3K)/protein kinase B (AKT) pathways and proved its therapeutic potential for ALD. HSYA treatment significantly decreases the triglyceride and low-density lipoprotein cholesterol (LDL-C) contents and increases the high-density lipoprotein cholesterol (HDL-C) in mice. HSYA interferes with the STAT3/NF-kB and PI3K/AKT/mechanistic target of rapamycin (mTOR) signaling pathways both in vitro and in vivo and efficiently suppresses ethanol-induced inflammation, lipid buildup, and hepatocyte death, and also strengthens the antioxidant defense system in the liver [144]. Lutein, a naturally occurring carotenoid and a xanthophyll, is found in green leafy vegetables, such as carrots, spinach, and kale. Lutein intervention markedly ameliorated the ALD in rats. In the liver, lutein lessens the occurrence of NF-κB and TLR4 proteins and also decreases the inflammatory cytokines, including TNF-α and IL-1β. It further restores the antioxidants in the liver. The hepatoprotective effect of lutein against alcohol-induced liver damage was claimed due to its anti-inflammatory and antioxidant properties [145]. Hence, immunological consequences of the NF-kB signaling axis have a significant role in the progression of ALD. The use of phytochemicals by targeting the NF-kB axis may be a promising approach to treating ALD. The diagrammatic representation of NF-кB and NLRP3 inflammasome signaling axis in ALD is shown in Figure 4.

### 2.6. MAPK Signaling Axis in ALD

Chronic ethanol consumption leads to liver injury through different signal transduction mechanisms, one of them being the mitogen-activated protein kinase signaling (MAPK) axis. It is involved in various cellular responses like differentiation, proliferation, and inflammation [146,147]. It consists of three different kinase cascades: (i) p42/44 MAPK, also termed extracellular signal-regulated kinase 1 and 2 (ERK1/2); (ii) p38 MAPK; and (iii) c-Jun N-terminal kinase (JNK), also called stress-activated protein kinase (SAPK). The literature suggests that alcohol-induced LPS release activates this pathway [147]. Acute alcohol exposure in the liver activates p42/44 MAPK in hepatocytes, whereas high exposure to ethanol leads to the release of endotoxins as PAMPs. As an extracellular signal regulator, ERK senses when the extracellular LPS release gets activated by it and causes transcription of early growth response-1 (Egr-1). The activation of both hepatocytes and Kupffer cells via the MAPK axis ultimately causes the production of TNF-α, instigating the inflammation [148,149]. Furthermore, acetaldehyde, a metabolite of ethanol, affects the HSC via activation of p38 MAPK and JNK, causing a further increase in the production of collagen and extracellular matrix deposition [150]. The generation of TNF-α and other cytokines will further activate other associated pathways and result in the production of more inflammatory mediators and tissue injury/necrosis in the liver. It has been identified that a few phytochemicals have the potential to reduce the risk associated with ALD via the inhibition of the MAPK signaling axis in the liver. A member of the hydroxycinnamic acid family, p-coumaric acid is a phenolic derivative. It is widely spread in plants and mushrooms and has different biological properties, like antioxidants and anti-inflammatory properties. In the ethanol-induced model, p-coumaric acid inhibits the phosphorylation of JNK, ERK, and p38 MAPK in the liver and exhibits hepatoprotective effects [151]. Sea buckthorn obtained from *Hippophae rhamnoides* is a deciduous shrub. It is edible and has medicinal properties. It helps in preventing alcoholic fatty liver disease by reducing the protein expression of p38 MAPK and p65 NF-kB [79]. The flavanone-7-O-glycoside, narirutin, is a compound of the flavone subclass. It is found in various fruits like oranges, tomatoes, beans, grapefruits, etc. It shows antioxidant and anti-inflammatory properties; it has been investigated for its hepatoprotective properties on alcohol-induced liver damage. It modulated the p38 MAPK signaling via binding to MAPK14, suppressed the mRNA level of *mapk14*, and exhibited hepatoprotective activity [152]. Similarly, glabridin, an isoflavone obtained from the lateral roots of *Glycyrrhiza glabra*, possesses hepatoprotective [153], anti-inflammatory, and antioxidant properties [154]. It participates in numerous pathways, including Wnt/β-catenin MAPK, Nrf2, and NF-kB pathways [153]. p38 MAPK, an important signaling pathway, is suppressed by glabridin, further ameliorating NF-kB-mediated inflammation and Nrf2-mediated oxidative stress in ethanol-fed C57BL/6J female mice [139]. Mechanistically, glabridin ameliorated ALD in mice via the p38 MAPK/Nrf2/NF-kB pathway. Narirutin, a flavanone-type polyphenolic compound, is mainly found abundantly in citrus peels, grapefruit, and oranges [155]. Acute exposure of zebrafish larvae to ethanol led to severe hepatic damage, and it successfully prevented alcohol-induced liver damage [152]. Elevations in mRNA genes linked to inflammation (TNF-α, IL-1β, and NF-κB) and oxidative stress markers were observed to be reversed upon treatment with narirutin. Liver injury and other liver illnesses are associated with abnormal p38 MAPK signaling [156]. Narirutin, by regulating the p38 MAPK pathway and targeting MAPK14, exerted a protective effect against ethanol-induced hepatic steatosis [152].

### 2.7. JAK-STAT Signaling Axis in ALD

In most of the alcohol-induced liver damage, cytokines are one of the essential factors that get activated and result in inflammation and tissue damage in the liver. These cytokines are released through different signaling pathways. Janus kinase/signal transducer and activator of transcription (JAK-STAT) signaling pathways are one of the pathways that are responsible for the transduction of various cytokines [157,158]. Along with controlling gene expressions, it is also involved in endoplasmic stress, apoptosis, autophagy, and other signaling processes [159,160]. Alcohol consumption leads to different physiological changes in the organs. They alter the gut microbiome, cause enteric dysbiosis, and produce or activate various DAMPs and PAMPs that will bind to different receptors like TLRs, TNFRs, and so on. LPS, when bound to these receptors, causes signal transduction to release multiple factors that cause damage and inflammation, in which the LPS-induced cytokines bind to their corresponding transmembrane receptors. This binding will further lead to the activation of JAK/STAT signaling, which causes altered gene expression. After ligand-receptor binding, activation of the pathway is achieved by causing a conformational change in the receptor. This change allows the JAK to enter the proximal receptor binding site to phosphorylate the tyrosine residue at the receptor in the cytoplasmic domain of the receptor. This process recruits the STAT molecule toward the receptor, which, in turn, phosphorylates and activates STAT. The activated STAT now dimerizes and then translocates to the nucleus. After entering the nucleus through the transcription factor, it leads to the transcription of the targeted genes, which propel the inflammation in the liver [161]. However, in different studies, it has been found that ethanol itself inhibits the JAK-STAT pathway, which contradicts its mechanism of JAK-STAT activation. In freshly isolated hepatocytes, acute ethanol blocks IL-6 or IFN-γ-induced STAT activation [162]. IL-6-activated STAT3 and IFN-induced STAT1 are inhibited by ethanol in monocytes. Hence, ethanol acts as an activator and inhibitor for JAK-STAT [163]. Further research is necessary to comprehend the detailed function of the JAK-STAT pathway in ALD. This dual behavior of ethanol may be due to differences in cell type, timing, dose of alcohol exposure, or specific cytokines involved in the signaling. Acute vs. chronic exposure, or systemic vs. liver-specific effects, might result in opposite outcomes. Therefore, a better understanding of the cellular context and temporal dynamics of ethanol action is critical, and future studies are needed to reconcile these differences. A list of phytochemicals/herbal products that modulate different immunological signaling pathways, along with cytokines and gut microbiome in ALD, is presented in Table 1.

Care must be taken to separate the action of individual phytochemicals from the action of crude plant extracts. Particular compounds, e.g., curcumin, quercetin, and resveratrol, act on clearly defined signal molecules—e.g., NF-κB, JAK-STAT, or MAPK—whereas plant extracts comprise a blend of bioactive constituents. These can have additive action or interact along multiple pathways. Thus, caution must be exercised in interpreting the mechanisms of action from extract-based studies, and wherever possible, results from purified phytochemicals should be given precedence for mechanistic insights.

## 3. Conclusions and Future Perspective

Bioactive compounds from nature have shown strong protective effects on the liver. This presents an exciting opportunity for therapeutic development. This is especially crucial when there is a pressing demand for new and effective treatments. These phytochemicals have minimal side effects and are relatively effective. Furthermore, they are readily available since they are derived from natural sources. However, they must be monitored for interactions with other metabolites or food and checked for physicochemical properties. The advancement of innovative treatments for individuals with ALD whose outlook is poor and lacking any interventions requires both translational and clinical studies. Additionally, getting accurate medicine based on multi-omics analysis and sex differences is necessary to conquer the obstacles and challenges to carrying out beneficial clinical studies on ALD patients. Accumulating evidence further indicates that phytochemicals, through a variety of immunological pathways, including cGAS-STING signaling, NLRP3 inflammasome, MAPK signaling, JAK-STAT signaling, enteric dysbiosis, and gut microbiome, lessen the alcohol-induced liver injuries. Hence, the development of phytomedicine-based therapy may be an alternative approach to mitigating ALD. In conclusion, immunological mechanisms play a crucial role in developing ALD. Despite promising preclinical results, the clinical translation of phytochemicals faces several challenges. These include poor bioavailability, lack of standardized dosing, variability in compound composition, and regulatory hurdles that complicate their approval as therapeutic agents. Targeting immunological mechanisms using phytochemicals or herbal medicine may find a place to counteract ALD.

## 4. Database Search

A comprehensive literature search was conducted in 2024, using PubMed, Google Scholar, Web of Science, and Science Direct databases. The search aimed to identify relevant studies published between 2005 and 2024 and employed the following search terms: “Alcohol-induced liver disease and Phytochemicals”, “Alcohol-induced liver disease and inflammation”; “Alcohol-induced liver disease and cGAS-STING signaling”; “Alcohol-induced liver disease and NLRP3 inflammasomes”; “Alcohol-induced liver disease and NF-κB signaling”; “Alcohol-induced liver disease and MAPK signaling”; and “Alcohol-induced liver disease and JAK-STAT signaling”.

## Figures and Tables

**Table 1:** A list of phytochemicals that modulate different immunological signaling pathways, along with cytokines and gut microbiome in alcohol-induced liver disease.

Name of Phytochemicals/Herbal Products	Preclinical Model	Mechanisms of Hepatoprotection	Ref.
Betaine (Dietary sources)	Short-term ethanol-fed C57BL/6J mice	↓ALT, ↓AST, ↓lipid accumulation, and **↑**SAM: SAH ratio	[164]
*Caulerpa lentillifera* (edible green seaweed) (green algae)	Chronic and binge alcohol-fed Wistar rats	**↑**AST, ↓AST, ↓GGT, ↓TLR4 pathway, and ↓ Gut dysbiosis	[47]
*Inula Britannica* (*Asteraceae*)	Chronic ethanol-fed C57BL/6J female mice	↓ALT, ↓AST, ↓liver TG, ↓TC Interferes SIRT1-AMPK/Nrf2/NF-κB axis, ↓ hepatic lipid buildup, **↑**antioxidant action, and ↓hepatic inflammation	[165]
Hydroxytyrosol	Chronic binge ethanol-fed male C57BL/6J mice	↓ALT, ↓AST, ↓liver TG, ↓TC, ↓LDL-C, interfere with STAT3/iNOS pathway and p-AKT/SREBP-1c pathway, and ↓hepatic inflammation	[166]
Daucosterol (*Sanchezia spesiosa*) (*Acanthaceae*)	Short-term chronic and binge ethanol-fed male C57BL/6J mice	↓ALT, ↓FFA, ↓liver TG, ↓p38/NF-κB/NLRP3, ↓hepatic lipid buildup, **↑**antioxidant action, and ↓hepatic inflammation	[122]
Ellagic acid (polyphenol)	Chronic alcohol-fed ICR mice	↓ALT, ↓AST, ↓ASP, ↓liver FFA, ↓liver TG, **↑**antioxidant action, ↓hepatic inflammation, improves gut microbiota	[167]
Withaferin A	Chronic binge ethanol-fed wild-type mice based on C57BL/6J	↓ALT, ↓AST, ↓liver TG ↓hepatic lipogenesis, and ↓hepatic lipid buildup	[168]
Glabiridin (Isoflavone) (*Glycyrrhiza glabra* L.)	Short-term chronic ethanol-fed C57BL/6J female mice.	↓ALT, ↓AST, ↓liver FFA, ↓liver TG, interfere with the p38 MAPK/Nrf2/NF-kB pathway, ↓ oxidative stress, and ↓hepatic inflammation	[139]
Nobiletin (Polymethoxylated flavone) (from citrus fruit peels)	Male C57BL/6N wild-type (WT) mice	↓ALT, ↓AST, ↓liver FFA, ↓liver cholesterol, interferes with NRF1-TFAM pathway, ↓hepatic inflammation, ↓oxidative stress, ↓ER stress, and ↓ apoptosis	[169]
Narirutin	EtOH-fed wild-type zebrafish larvae	↓ALT, ↓AST, interfere with p38-MAPK pathway, ↓hepatic inflammation, ↓oxidative stress, ↓ER stress, and ↓lipid accumulation	[152]
Hydroxysafflor yellow A	Chronic and binge alcohol-fed C57BL/6J male mice.	↓ALT, ↓AST, ↓LDL, **↑**HDL, ↓liver TG, interfere with STAT3/NF-kB and PI3K/AKT/mTOR pathways, ↓hepatic inflammation, ↓oxidative stress, ↓ER stress, ↓lipid accumulation, and ↓hepatocyte apoptosis	[144]
Ginsenoside Rk1	Alcohol-fed wild-type zebrafish	↓liver lipid content, ↓liver TG Interferes with NF-kB pathway, and ↓hepatic inflammation	[82]
Ginsenoside Rk2 (*Panax notoginseng*) (*Araliaceae*)	Ethanol-fed C57BL/6J male mice	↓ALT, ↓AST, interfere with Nrf2/HO-1 pathway, ↓oxidative stress, block the NF-kB/NLRP3 pathway, and ↓hepatic inflammation	[124]
Astragaloside (*Astragalus membranaceus*)	Chronic and binge alcohol-fed SD rats	↓ALT, ↓AST, ↓LDL, **↑**HDL, ↓liver lipid content, ↓NF-kB pathway, ↓hepatic inflammation, ↓oxidative stress, ↓ER stress, ↓lipid peroxidation, and ↓hepatocyte apoptosis	[170]
*Diammonium glycyrrhizinate*	Chronic and binge alcohol-fed C57BL/6J male mice	↓ALT, ↓AST, ↓liver TG, ↓serum TG, ↓DDX5/STAT1 axis, ↓hepatic lipid buildup, **↑**antioxidant action, and ↓hepatic inflammation	[171]
Scutellarin (*Erigeron breviscapus)* (Asteraceae)	Binge alcohol-fed C57BL/6J male mice	↓ALT, ↓AST, interfere Nrf2/HO-1 pathway & AKT, p38 MAPK/NF-kB pathway, and ↓hepatic inflammation	[126]
Lutein	Chronic and binge alcohol-fed male Wistar rats	↓ALT, ↓AST, ↓GGT, ↓serum TG, **↑**Nrf2/HO-1 pathway, ↓TLR4/NF-kB pathway, ↓hepatic inflammation, and ↓ oxidative stress	[145]
Myricetin	Chronic and binge alcohol-fed male Wistar rats	↓ALT, ↓AST, ↓LDH, ↓lipid peroxidation, interfere with the NF-kB pathway, and ↓hepatic inflammation	[83]
*Allium ochotense* (Amaryllidaceae)	Alcohol-fed C57BL/6J mice	↓CHL, ↓TG, ↓LDL, and ↓lipid peroxidation	[172]
*Schisandra sphenanthera* (Magnoliaceae)	Chronic alcohol-fed male Sprague Dawley rats	↓ALT, ↓AST, ↓ADH, ↓ALDH, interfere with the PI3K-AKT pathway, ↓hepatic inflammation, and ↓oxidative stress	[173]
Oroxylin A, obtained from *Scutellaria biacalensis*, is a flavonoid compound.	CCl-4 induced mice model, 8 weeks	Oroxylin A inhibits the cGAS-STING pathway and induces the ferritinophagy of HSC	[102]
Extract of LanGui tea, a flavonoid-rich formulation containing *Gynostemma pentaphyllum*, *Cinnamomum cassia*, and *Ampelopsis grossedentata*.	Alcohol-induced male C57BL/6 mice model	Inhibits NLRP3 signaling and decreases the generation of IL-1β	[116]
Quercetin, a polyphenol	Alcohol-induced male Wistar rat model	It enhances the occurrence of HO-1 and IL-10 and, thus, inhibits NLRP3 inflammasome activation	[117]
Cannabidiol, extracted from marijuana plants	Ethanol plus high-fat diet male C57B/6J mice model	Inhibits the recruitment of macrophages, and thus, it leads to the inhibition of the NLRP3-pyroptosis pathway	[118]
Taxifolin, a dihydroflavone found in onions and milk thistle	Alcohol-induced male C57BL/6 mice model	Inhibits P2X7R-signaling IL-1β secretion by inactivating the NLRP3 inflammasome pathway	[119]
p-coumaric acid, a hydroxycinnamic acid family	Ethanol-induced male Wistar rat model, 28 days	Inhibits phosphorylation of JNK, p38 MAP kinase, and ERK.	[151]
Sea buckthorn (*Hippophae rhamnoides*)	Male pathogen-free KM mice model	Reduces the expression of MAPK p38 protein and inflammatory cytokines	[79]
Narirutin, a flavone type flavonoid	Alcohol-induced zebrafish larvae model	Modulated the p38 MAPK signaling via binding to the MAPK14 and also suppressed the mRNA level of *mapk14*	[152]
Europinidin, obtained from *Plumbago europea*	Ethanol-induced male Wistar rat model	Inhibits pro-inflammatory cytokines and genes via inhibiting NF-kB initiation	[135]
Curcumin, obtained from *Curcuma longa*	Alcohol-induced rat, mouse model	It regulates the IkBα-NF-kB pathway to further decrease inflammation	[136]
Artemisinin, isolated from *Artemisia annua*, is a sesquiterpene lactone	Alcohol-induced male KM mice model	Inhibits NF-кB activation and reduces the expression of the inflammatory cytokines	[137]
Grape leaf extract, a phenolic compound isolated from leaves of the plant *Vitis vinifera*	Ethanol-induced male Sprague Dawley rat model	Suppress ethanol-induced NF-кB p65 subunit and TNF-α	[138]
Glabridin, an isoflavone obtained from licorice root	Ethanol-induced C57BL/6 female mice model	Decreases the nuclear translocation of NF-кB	[139]
Genistein, isolated from *Hydrocotyle sibthorpioides* Lam.	Alcohol-induced male SPF-Wistar rat model, 24 weeks	Reduces the DNA binding activity of NF-кB and downregulates its activity	[140]
Apigenin (4′, 5, 7-trihydroxyflavone), a flavonoid compound	Alcohol-induced male KM-mice model, 30 days	Increases expression of PPARα, downregulates the NF-kB signaling	[141]
Combination of epigallocatechin-3-gallate, taurine, and genistein	Alcohol-induced rat liver fibrosis model, 24 weeks	Restricted the production and secretion of the inflammatory cytokines like IL-6, TNF-α	[81]
Epigallocatechin-3-gallate, a phenolic compound	Alcohol-induced female Sprague-Dawley rat model, 5 or more weeks	Inhibits gut leakiness and reduces endotoxemia	[43]
Lychee (*Litchi chinensis* Sonn)	Alcohol-induced male C57Bl/6 mice model, 8 weeks	Lychee pulp extract increases the production of mucus-protecting proteins and intestinal tight junction proteins and lowers the number of endotoxins in the blood.	[44]
A mixture of *Ginkgo biloba* and *Rosa roxburghii*	Alcohol-induced male Sprague Dawley rat model, 8 weeks	They restore tight junctions, hence protecting the intestinal barrier dysfunction	[45]
Rice bran phenolic extract	Alcohol-induced C57BL/6 mice model, 8 weeks	Its supplementation decreases pathogenic bacteria in the gut and protects the intestinal barrier, function, and permeability from alcohol.	[46]

↓ indicates decreased, **↑** indicates increased.

## Data Availability

Not Applicable.

## Abbreviations

The following abbreviations are used in this manuscript:

AFL	Alcoholic fatty liver
ALD	Alcohol-induced liver disease
APCs	Antigen-presenting cells
ASC	Apoptosis-associated speck-like protein
cGAS	Cyclic guanosine monophosphate-adenosine monophosphate (cGAMP) synthase
DAMPs	Damage-associated molecular patterns
EGCG	Epigallocatechin-3-gallate
ERK	Extracellular signal-regulated kinase
HSCs	Hepatic stellate cells
IFN	Interferon
IFR	Interferon regulatory factor
ILs	Interleukins
JAK-STAT	Janus kinase/signal transducer and activator of transcription
NK	c-Jun N-terminal kinase
LPS	Lipopolysaccharide
MAPK	Mitogen-activated protein kinase
NF-kB	Nuclear factor–kappa B
NKT	Natural killer T cells
NLRP3	NOD-like receptor family pyrin domain containing 3
PAMPs	Pathogen-associated molecular patterns
PRR	Pattern recognition receptor
ROS	Reactive oxygen species
STING	Stimulator of interferon gene
TGF-β	Transforming growth factor-β
TLR	Toll-like receptor
TNF	Tumor necrosis factor
WHO	World Health Organization
